# Auxin response factor 6A regulates photosynthesis, sugar accumulation, and fruit development in tomato

**DOI:** 10.1038/s41438-019-0167-x

**Published:** 2019-07-11

**Authors:** Yujin Yuan, Xin Xu, Zehao Gong, Yuwei Tang, Mengbo Wu, Fang Yan, Xiaolan Zhang, Qian Zhang, Fengqing Yang, Xiaowei Hu, Qichen Yang, Yingqing Luo, Lihua Mei, Wenfa Zhang, Cai-Zhong Jiang, Wangjin Lu, Zhengguo Li, Wei Deng

**Affiliations:** 1 Key Laboratory of Plant Hormones and Development Regulation of Chongqing, School of Life Sciences, Chongqing University, 401331 Chongqing, China; 2 School of Chemistry and Chemical Engineering, Chongqing University, 400044 Chongqing, China; 3 College of Basic Science, Tianjin Agricultural University, 300384 Tianjin, China; 4 Department of Plant Sciences, University of California, Davis, CA 95616 USA; 5 Crops Pathology and Genetics Research Unit, United States Department of Agriculture, Agricultural Research Service, Davis, CA 95616 USA; 6 State Key Laboratory for Conservation and Utilization of Subtropical Agro-bioresources/Guangdong Provincial Key Laboratory of Postharvest Science of Fruits and Vegetables, College of Horticulture, South China Agricultural University, 510642 Guangzhou, China

**Keywords:** Plant molecular biology, Agricultural genetics

## Abstract

Auxin response factors (ARFs) are involved in auxin-mediated transcriptional regulation in plants. In this study, we performed functional characterization of SlARF6A in tomato. SlARF6A is located in the nucleus and exhibits transcriptional activator activity. Overexpression of *SlARF6A* increased chlorophyll contents in the fruits and leaves of tomato plants, whereas downregulation of *SlARF6A* decreased chlorophyll contents compared with those of wild-type (WT) plants. Analysis of chloroplasts using transmission electron microscopy indicated increased sizes of chloroplasts in *SlARF6A*-overexpressing plants and decreased numbers of chloroplasts in *SlARF6A*-downregulated plants. Overexpression of *SlARF6A* increased the photosynthesis rate and accumulation of starch and soluble sugars, whereas knockdown of *SlARF6A* resulted in opposite phenotypes in tomato leaves and fruits. RNA-sequence analysis showed that regulation of *SlARF6A* expression altered the expression of genes involved in chlorophyll metabolism, photosynthesis and sugar metabolism. *SlARF6A* directly bound to the promoters of *SlGLK1*, *CAB*, and *RbcS* genes and positively regulated the expression of these genes. Overexpression of *SlARF6A* also inhibited fruit ripening and ethylene production, whereas downregulation of *SlARF6A* increased fruit ripening and ethylene production. *SlARF6A* directly bound to the *SAMS1* promoter and negatively regulated *SAMS1* expression. Taken together, these results expand our understanding of ARFs with regard to photosynthesis, sugar accumulation and fruit development and provide a potential target for genetic engineering to improve fruit nutrition in horticulture crops.

## Introduction

Tomato is the world’s second largest vegetable crop rich in nutrients^1^. Tomato fruit development includes three stages^2^. The first stage is characterized by an increase in cell number and starch accumulation, followed by cell enlargement with starch degradation and soluble sugar accumulation in the second stage^3^. Fruit ripening is the last stage, associated with the accumulation of soluble sugars, carotenoids, organic acids, and volatile organic compounds in fruits^1^.

The chlorophyll accumulation and photosynthetic activity of green fruits influence the nutritional components and flavor of ripening tomato fruits^4^. Some genes have been reported to affect chlorophyll accumulation, chloroplast development and fruit quality. As negative regulators, DE-ETIOLATED 1/high pigment 2 (DET1/hp2) and UV-DAMAGED DNA-BINDING PROTEIN 1/high pigment 1 (DDB1/hp1) are involved in chloroplast formation and chlorophyll accumulation in tomato fruits^5,6^. The tomato GOLDEN2-LIKE transcription factors SlGLK1 and SlGLK2 play an important role in chloroplast formation and chlorophyll accumulation^7^. Evidence suggests that the *SlGLK2* gene is predominantly expressed in fruits and that the latitudinal gradient of *SlGLK2* expression influences the production of unevenly colored tomato fruits^8^. Overexpression of the *APRR2-LIKE* gene, the closest homolog of *SlGLK2*, increased the size and number of chloroplasts and enhanced chlorophyll accumulation in green tomato fruits^9^. TKN2 and TKN4, two Class I KNOTTED1-LIKE HOMEOBOX (KNOX) proteins, act as transcriptional activators of *SlGLK2* and *APRR2-LIKE* genes to promote chloroplast development in tomato fruits^4^. BEL1-LIKE HOMEODOMAIN11 (SlBEL11) also plays an important role in chlorophyll synthesis and chloroplast development in tomato fruits^10^.

The ripening of tomato is mainly regulated by the ethylene pathway and many transcription factors^1,11,12^. In the ethylene biosynthetic pathway, S-adenosylmethionine synthetase (SAMS) catalyzes the reaction of ATP and methionine to form S-adenosyl-L-methionine (SAM)^13^. 1-Aminocyclopropane-1-carboxylic acid (ACC) synthase (ACS) and ACC oxidase (ACO) catalyze the conversion of SAM to ACC and of ACC to ethylene, respectively. The MADS box gene *RIPENING INHIBITOR* (*RIN*) controls the early phase of ripening and ethylene production via transcriptional regulation of ACSs and ACOs^14^. The other ripening regulators affecting ethylene production also include the NAC transcription factor NOR, the SQUAMOSA PROMOTER BINDING protein CNR, the ethylene response factor ERF B3, the AP2/ERF member AP2a, and several MADS box proteins, such as TDR4/SlFUL1, SlFUL2, SlMADS1, TAGL1, and TAG1^15–21^.

Auxin is an important phytohormone involved in flower fertilization, fruit setting, fruit initiation and development^22^. Auxin is also essential in the regulation of cell division and expansion, controlling final fruit size^23^. Auxin modulates plant development through transcriptional regulation of auxin-responsive genes, which is primarily mediated by two gene families: the short-lived nuclear protein Aux/IAA family and auxin response factors (ARFs)^1,24–26^. Most ARFs have an N-terminal DNA-binding domain (B3) required for transcriptional regulation of auxin response genes, a middle region functioning as a repression domain (RD) or activation domain (AD), and a C-terminal dimerization domain (CTD) involved in the formation of homodimers or heterodimers^27^. ARFs can act as either an activator or a repressor of the transcription of auxin-responsive genes^27^. Numerous studies have indicated that ARFs are involved in many tomato developmental processes^27–32^. *SlARF4* negatively regulates chlorophyll accumulation and starch biosynthesis in tomato fruit^33,34^. Our previous study showed that *SlARF10* positively regulated chlorophyll accumulation via direct activation of the expression of *SlGLK1*^35^. Downregulation of *ARF6* and *ARF8* by overexpression of *Arabidopsis* microRNA167 results in the failure of pollen germination on the stigma surface and/or growth through the style in tomato^36^. However, the function of *SlARF6* in the regulation of fruit development is still not well understood. In this study, *SlARF6A* was found to be involved in photosynthesis, sugar accumulation and fruit development in tomato. Our data demonstrate that SlARF6A plays an important role in the regulation of fruit quality and development.

## Results

### Sequence and expression analysis of *SlARF6A* gene and subcellular localization and transcriptional activity of SlARF6A protein

The *SlARF6A* gene has an open reading frame of 2608 bp encoding a putative protein of 869 amino acids. Amino acid sequence analysis revealed that, like SlARF7 and SlARF8, which have typical conserved ARF domains, SlARF6A protein also contained B3-DNA, ARF, and AUX/IAA binding domains (Fig. S1). A phylogenetic tree was constructed to gain insight into the phylogenetic relationship among ARF proteins in Arabidopsis and tomato. ARFs were divided into four major classes: I, II, III, and VI^37^. SlARF6A along with SlARF6B and AtARF6 were grouped into subclass IIa and are closely related to AtARF8 and SlARF8 (Fig. S2), indicating possible functional similarity among them.

**Fig. 1:**
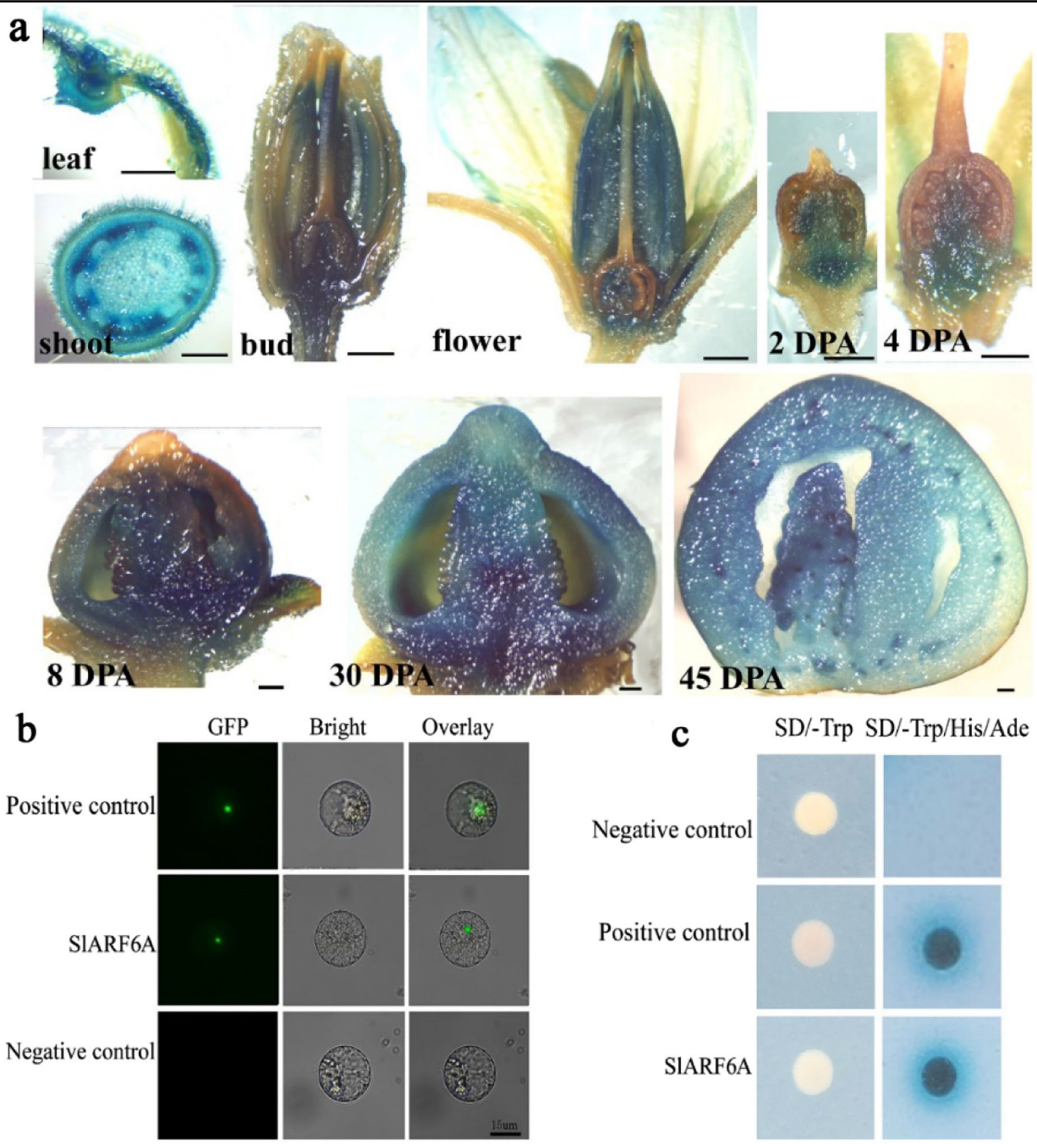
Molecular properties of SlARF6A. **a** Expression pattern of *SlARF6A* revealed by the expression of the *GUS* reporter gene driven by the *SlARF6A* promoter. Gus staining was conducted using leaf, shoot, bud, flower, and fruit tissues from transgenic plants at 2, 4, 8, 30, and 45 days post anthesis (DPA). The bar is 1 mm. **b** Subcellular localization analysis of SlARF6A protein. The SlARF6A-GFP fusion protein and GFP-positive and GFP-negative controls (PCXDG-GFP) were transiently expressed in tobacco (*Nicotiana benthamiana*) leaves. Images were taken in a dark field for green fluorescence, while the outline of the cells and the merged image were recorded in a bright field. The bar is 15 μm. **c** Transcriptional activation activity of SlARF6A protein. The pGBKT7-*SlARF6A* fusion vector, negative control (Empty pGBKT7 vector) and positive control were transformed into Y2H gold yeast cells. The yeast cells were cultivated on medium without tryptophan (SD-Trp) or without tryptophan, histidine, and adenine (SD-Trp/His/Ade)

To determine the expression pattern of *SlARF6A* in planta, a transcriptional fusion was constructed between the *SlARF6A* promoter and the GUS reporter gene. GUS staining in the transgenic tomato plants was detected in leaves, stems, buds, flowers, and fruits at different developmental stages, an indication of the ubiquitous expression of *SlARF6A* in all tissues tested. The GUS staining was weak in the early fruits at 2 and 4 days post anthesis (DPA) but became strong at 8, 30 and 45 DPA (Fig. 1a), suggesting possible roles of *SlARF6A* in the development of tomato fruits.

To examine its subcellular localization in plants, SlARF6A was fused to GFP and transferred into tobacco protoplasts. Fluorescence microscopy analysis revealed that SlARF6A was specifically localized in the nuclei (Fig. 1b). A GAL4-responsive reporter system in yeast was employed to reveal the transcriptional activity of SlARF6A. SlARF6A was fused to GAL4-BD (DNA binding domain) to form a pGBKT7-SlARF6A fusion plasmid and subsequently transformed into yeast. Yeast transformants harboring the pGBKT7-SlARF6A construct grew well in the medium lacking Trp, His, and Ade (SD-W/H/A), while the yeasts transformed with pGBKT7 vector alone (negative control) could not (Fig. 1c). Assessing transcriptional activity revealed that SlARF6A is a transcriptional activator.

### *SlARF6A* is involved in chlorophyll accumulation and chloroplast development in tomato

**Fig. 2:**
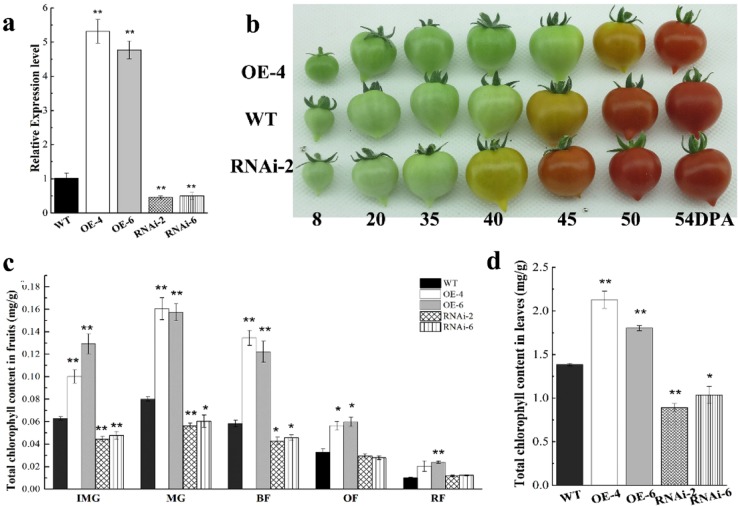
Generation of SlARF6A transgenic plants, chlorophyll accumulation, and chloroplast observation in SlARF6A transgenic plants. **a** qRT-PCR analysis of the expression of *SlARF6A* in transgenic lines. The data represent the mean ± SD of four biological replicates. **b** Fruit phenotypes. WT, wild-type plants; OE, *SlARF6A* overexpression lines; RNAi, *SlARF6A* RNAi lines. DPA, days post anthesis. **c** Chlorophyll contents in fruits of OE-SlARF6A and RNAi-SlARF6A plants. **d** Chlorophyll contents in leaves of OE-SlARF6A and RNAi-SlARF6A plants. The data represent the mean ± SD of three biological replicates. “*” and “**” are significant differences between transgenic and WT plants at the *P* < 0.05 and *P* < 0.01 levels, respectively, as determined by *t*-test

To elucidate the physiological significance of the *SlARF6A* gene in fruit development, upregulated and downregulated transgenic lines corresponding to independent transformation events were generated in tomato plants. qRT-PCR was used to evaluate the expression level of *SlARF6A* in all transgenic lines. Compared with the level in the wild type (WT), the expression level of *SlARF6A* was decreased in RNAi 2 and 6 plants (Fig. 2a) but increased in OE-4 and 6 plants (Fig. 2a).

**Fig. 3:**
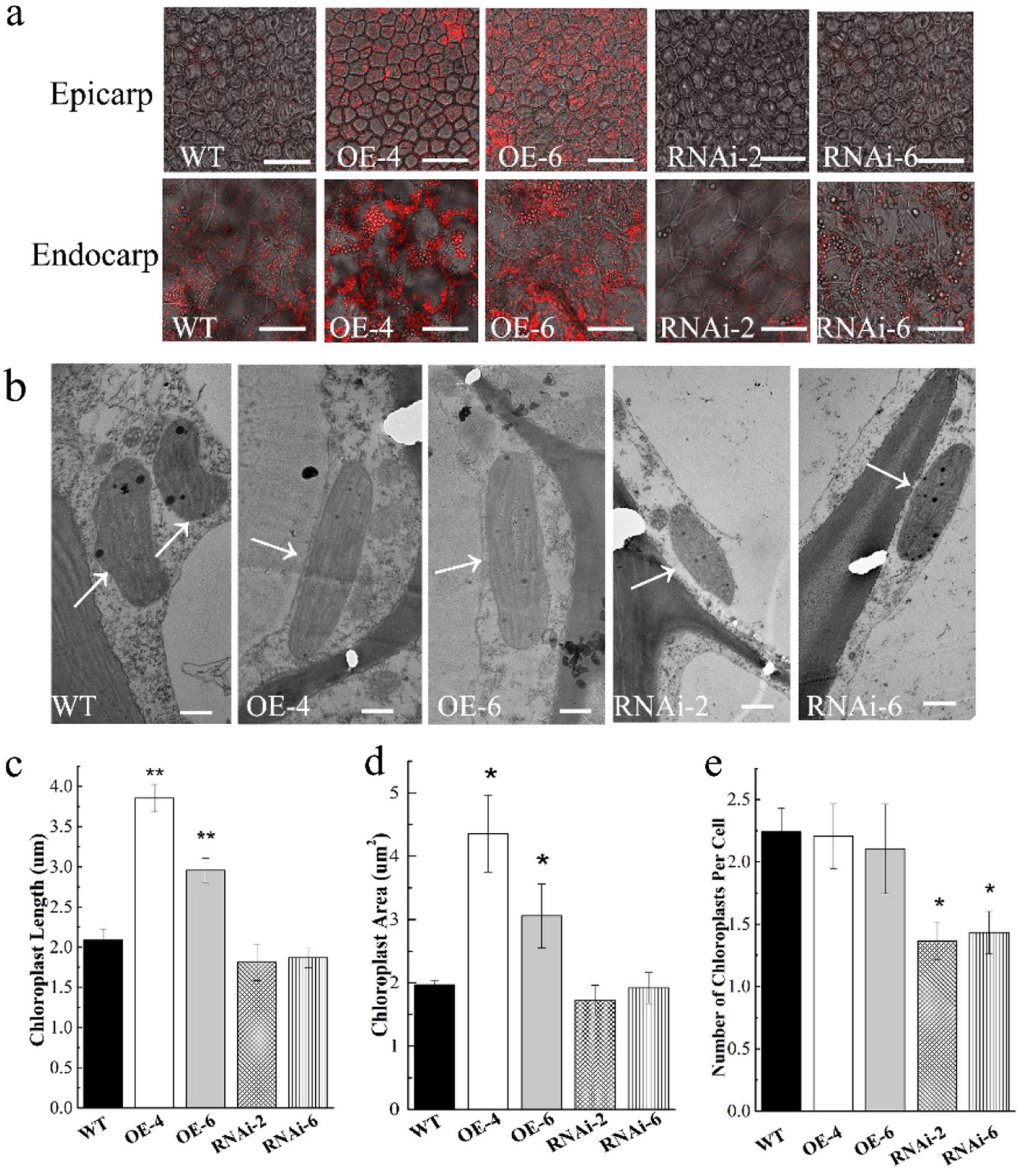
Autofluorescence and TEM analysis in transgenic and WT fruits. **a** Autofluorescence of chlorophylls in the pericarp of tomato fruits, as determined by confocal laser scanning microscopy. The bar is 10 μm. **b** TEM analysis of chloroplasts in transgenic and WT fruits. The bar is 10 μm. White arrows indicate chloroplasts. **c** Chloroplast size analysis. **d** Chloroplast length analysis. **e** Number of chloroplasts per cell. The data represent the mean ± SD of three biological replicates. “*” indicates significant differences between transgenic and WT plants at *P* < 0.05 as determined by *t*-test

It is noteworthy that altered *SlARF6A* expression led to a dramatic change in chlorophyll accumulation in transgenic lines. Compared with WT plants, the OE-SlARF6A plants had dark-green fruits at the green fruit stage, whereas the RNAi-SlARF6A plants had light-green fruits (Fig. 2b). The impact of altered *SlARF6A* expression on chlorophyll accumulation was analyzed by measuring the chlorophyll content in fruits and leaves. The *SlARF6A* overexpression lines possessed greater accumulation of chlorophyll in the fruits at immature green, mature green, breaker, and orange stages, whereas the RNAi lines had lower chlorophyll accumulation in the fruits at immature green and mature green stages than the WT plants (Fig. 2c). In leaves, the upregulated and downregulated *SlARF6A* transgenic lines possessed higher and lower chlorophyll levels, respectively, than the WT plants (Fig. 2d). Then, chlorophyll autofluorescence in the pericarp was detected using confocal laser scanning microscopy. OE-SlARF6A plants had stronger chlorophyll autofluorescence, while the RNAi-SlARF6A lines had weaker chlorophyll autofluorescence in both epicarp and endocarp tissues compared with that of the WT plants (Fig. 3a). Then, the chloroplasts were observed using a transmission electron microscope (TEM). The growth of individual chloroplasts in OE-SlARF6A fruits was obviously promoted, with a significant increase in size and length (Fig. 3b). However, the number of chloroplasts per cell in OE-SlARF6A fruits was the same as that in the WT plants. For the RNAi-SlARF6A lines, the number of chloroplasts per cell was obviously decreased, but the size of individual chloroplasts was not changed (Fig. 3c–e).

### *SlARF6A* positively affects photosynthesis and photosynthate accumulation in tomato

**Fig. 4:**
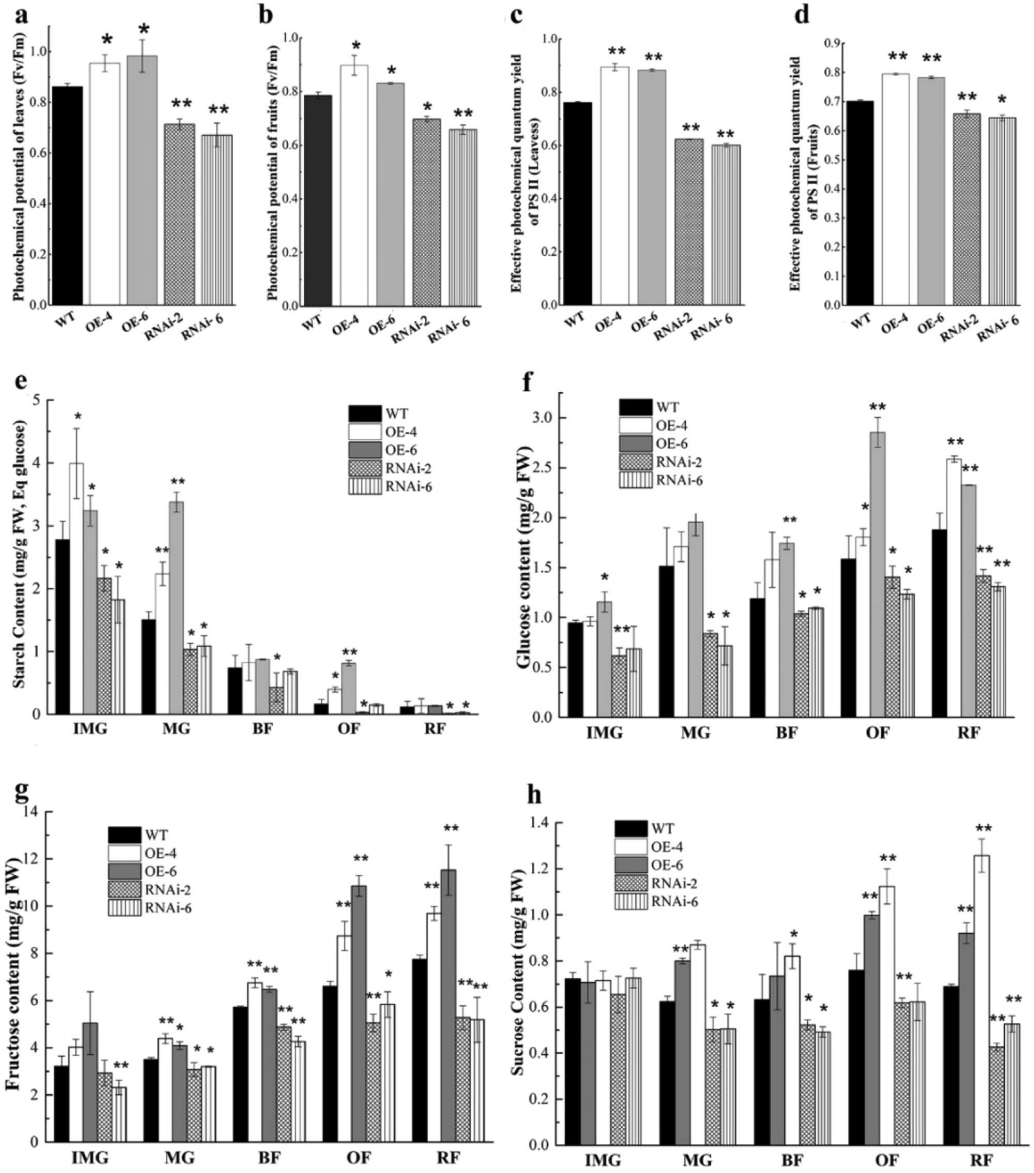
Photochemical potential of SlARF6A transgenic plants and accumulation of photosynthetic substances in transgenic fruits. **a** Photochemical potential in leaves. **b** Photochemical potential in fruits. **c** Effective photochemical quantum yield of PS II in leaves. d, Effective photochemical quantum yield of PS II in fruits. **e**–**h** demonstrate the contents of starch (**e**), glucose (**f**), fructose (**g**), and sucrose (**h**) in transgenic plants, respectively. The data represent the mean ± SD of three biological replicates. “*” and “**” indicate significant differences between the transgenic and WT plants at *P* < 0.05 and *P* < 0.01, respectively, as determined by *t*-test

The dark-green phenotype and associated increased chlorophyll content may potentially lead to enhanced photosynthetic performance in tomato plants. The photosynthetic performance in leaves and fruits of *SlARF6A* transgenic lines was measured. In both leaves and green fruits, the photochemical potential was elevated in OE-SlARF6A lines, whereas the value was decreased in RNAi-SlARF6A plants compared with the WT plants (Fig. 4a, b). The effective photochemical quantum yield of PSII in OE-SlARF6A lines was higher than that of the WT plants in both leaves and fruits, while the values for RNAi-SlARF6A plants were lower than that for the WT plants in both leaves and fruits (Fig. 4c, d). Thus, the *SlARF6A* gene positively affects photosynthesis in the fruits and leaves of tomato plants.

Sugars are the major products of photosynthesis, so it is essential to evaluate whether the altered chlorophyll level and photosynthetic performance in *SlARF6A* plants result in altered sugar accumulation. As shown in Fig. 4e, starch levels decreased rapidly throughout fruit development in the transgenic and WT plants. The starch content in OE-SlARF6A fruits was much higher than that in WT fruits at green fruit stages, whereas the starch content in RNAi-SlARF6A fruits was much lower than that in the WT fruits at green stages (Fig. 4e). These data demonstrated that the *SlARF6A* gene positively affects starch accumulation during green fruit development.

It is well established that starch degradation is the dominant source of soluble sugars in fruits. The contents of fructose, glucose and sucrose were analyzed in *SlARF6A* transgenic plants. The levels of glucose, fructose and sucrose were significantly higher in the OE-SlARF6A fruits than in the WT fruits, particularly at the orange and red fruit stages (Fig. 4f–h). Compared with the WT fruits, the RNAi-SlARF6A fruits exhibited obviously decreased contents in glucose, fructose and sucrose (Fig. 4f–h). Our data indicated that the *SlARF6A* gene positively affects the levels of glucose, fructose and sucrose during fruit development.

### *SlARF6A* is involved in fruit ripening and ethylene production in tomato

**Fig. 5:**
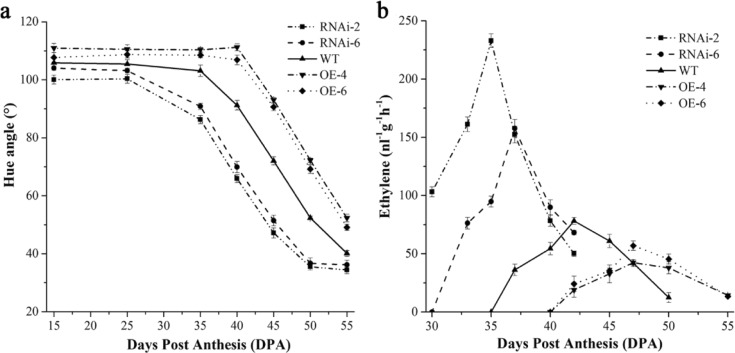
Altered fruit ripening features of SlARF6A transgenic plants. **a** Changes in hue angle in the WT and *SlARF6A* transgenic plants. **b** Ethylene production of the WT and *SlARF6A* transgenic plants at different ripening stages indicated as days post anthesis (DPA). The data represent the means of at least 10 individual fruits. Vertical bars represent SD. In WT plants, 35 DPA corresponds to the mature green (MG) stage, and 40 DPA corresponds to the breaker (BR) stage. In OE-SlARF6A plants, 40 DPA corresponds to the mature green (MG) stage, and 45 DPA corresponds to the breaker (BR) stage. In RNAi-SlARF6A plants, 30 DPA corresponds to the mature green (MG) stage, and 35 DPA corresponds to the breaker (BR) stage

The *SlARF6A* transgenic plants also exhibited different ripening of fruits than the WT plants. Downregulation of *SlARF6A* accelerated fruit ripening, with the breaker stage occurring 5 days sooner than that in the WT plants (Fig. 2b), while overexpression delayed the breaker stage by 5 days compared with that of the WT plants (Fig. 2b). The assessment of color change via measurement of the evolution of hue angle values further confirmed the difference between the *SlARF6A* transgenic lines and WT plants throughout the ripening process (Fig. 5a). The ethylene production was measured using a GC method. When compared with that of the WT plants, the ethylene production of RNAi-SlARF6A plants showed a dramatic induction of ~2-fold and 4-fold at the breaker stage and remained at high levels for 2 and 3 days after the breaker stage, while that of overexpressed lines was inhibited at the breaker stage and remained at low levels for 5 days after the breaker stage compared with the levels in the WT plants (Fig. 5b).

### Regulation of *SlARF6A* expression alters the expression of genes involved in chlorophyll metabolism, photosynthesis and sugar metabolism

**Fig. 6:**
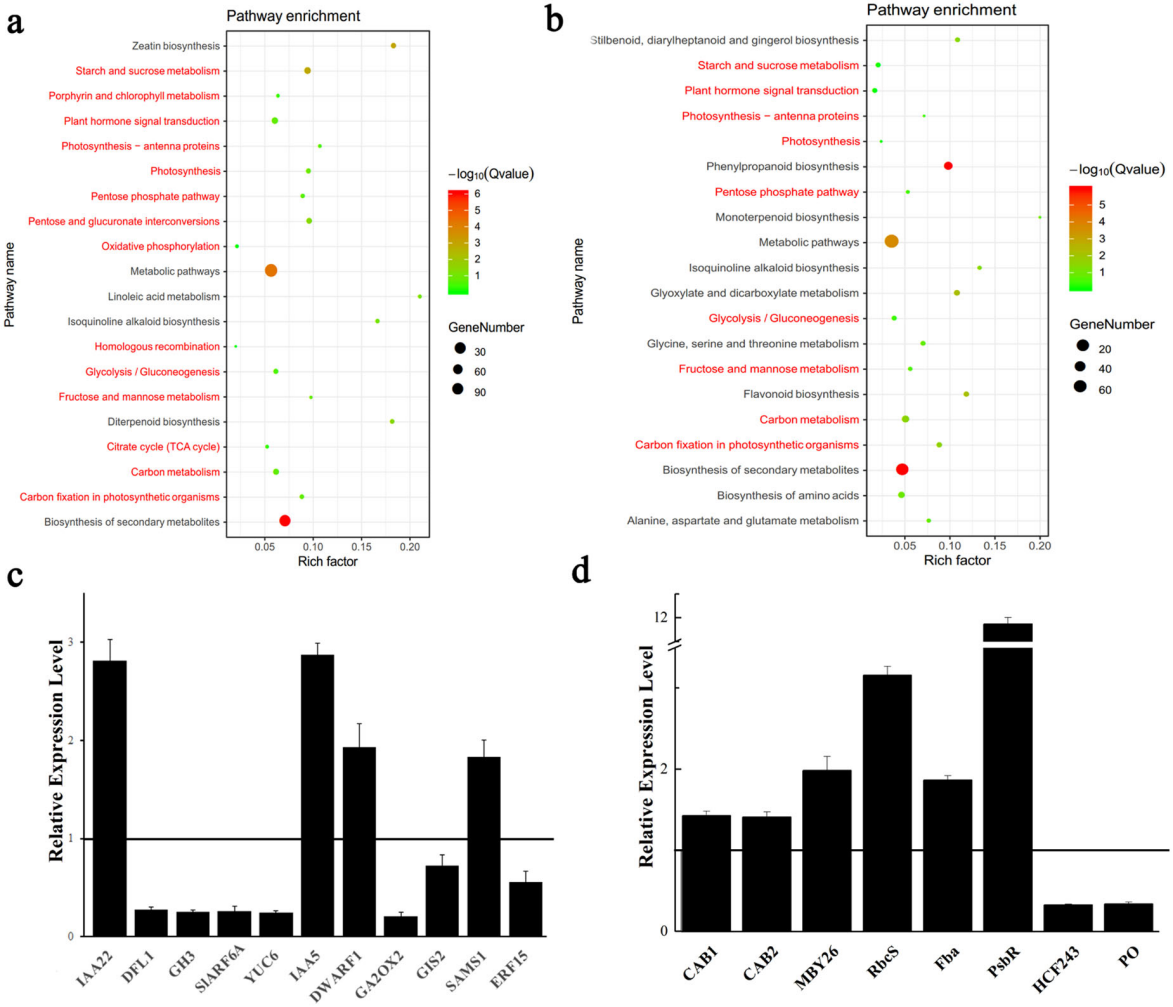
RNA-Seq analysis of SlARF6A transgenic plants. **a** Functional categories of differentially expressed genes (DEGs) between WT and RNAi-SlARF6A plants. **b** Functional categories of differentially expressed genes (DEGs) between WT and OE-SlARF6A plants. **c** Transcript levels of the genes identified from the RNA-Seq analysis were validated by qRT-PCR in the RNAi-*SlARF6A* plants (**c**) and OE-*SlARF6A* plants (**d**). The solid line indicates relative expression levels in the WT. The data represent the mean ± SD of four biological replicates

To investigate the molecular mechanism of chlorophyll accumulation, photosynthesis and fruit ripening in *SlARF6A* transgenic plants, RNA-sequencing (RNA-Seq) was conducted to analyze the differentially expressed genes (DEGs) in OE-SlARF6A and RNAi-SlARF6A plants. Under the criterion of a false discovery rate (FDR) < 0.05, 591 upregulated and 508 downregulated DEGs were identified in 4 DPA ovaries of RNAi-SlARF6A plants, and 254 upregulated and 424 downregulated DEGs were identified in 35 DPA fruits of OE-SlARF6A plants (Table S1). GO function and pathway enrichment analyses showed that knockdown of *SlARF6A* affected multiple metabolic pathways, including those of porphyrin and chlorophyll metabolism, photosynthesis, photosynthesis-antenna proteins, carbon fixation, starch and sucrose metabolism, fructose and mannose metabolism, and plant hormone signal transduction (Fig. 6a, Table S2). Overexpression of *SlARF6A* also affected metabolic pathways, including those of photosynthesis, photosynthesis-antenna proteins, carbon fixation, starch and sucrose metabolism, fructose and mannose metabolism, and plant hormone signal transduction (Fig. 6b, Table S3). The expression of two genes encoding chlorophyll A/B binding protein (CAB1 and CAB2) (Solyc02g070950 and Solyc02g071010) was induced in OE-SlARF6A plants. The expression of a gene encoding ribulose bisphosphate carboxylase small chain (RbcS) (Solyc02g085950) was upregulated in OE-SlARF6A plants. Moreover, the expression of a gene encoding SAM synthetase 1 (SAMS1) (Solyc12g099000), which is involved in ethylene biosynthesis, was induced in RNAi-SlARF6A plants. Analysis of the RNA-Seq data also showed that among tomato ARF family genes, only *SlARF6A* was downregulated in RNAi-SlARF6A plants, indicating the specific knockdown of SlARF6A by the RNAi method. To validate the RNA-Seq results, 11 DEGs in RNAi-SlARF6A plants and 8 DEGs in OE-SlARF6A plants were selected for qRT-PCR analysis, and the results were in accordance with the data from RNA-Seq (Fig. 6c, d), which showed that the results from the RNA-Seq were reproducible and reliable.

### SlARF6A targets the promoters of *CAB*, *RbcS* and *SlGLK1* genes and positively regulates the expression of these genes

**Fig. 7:**
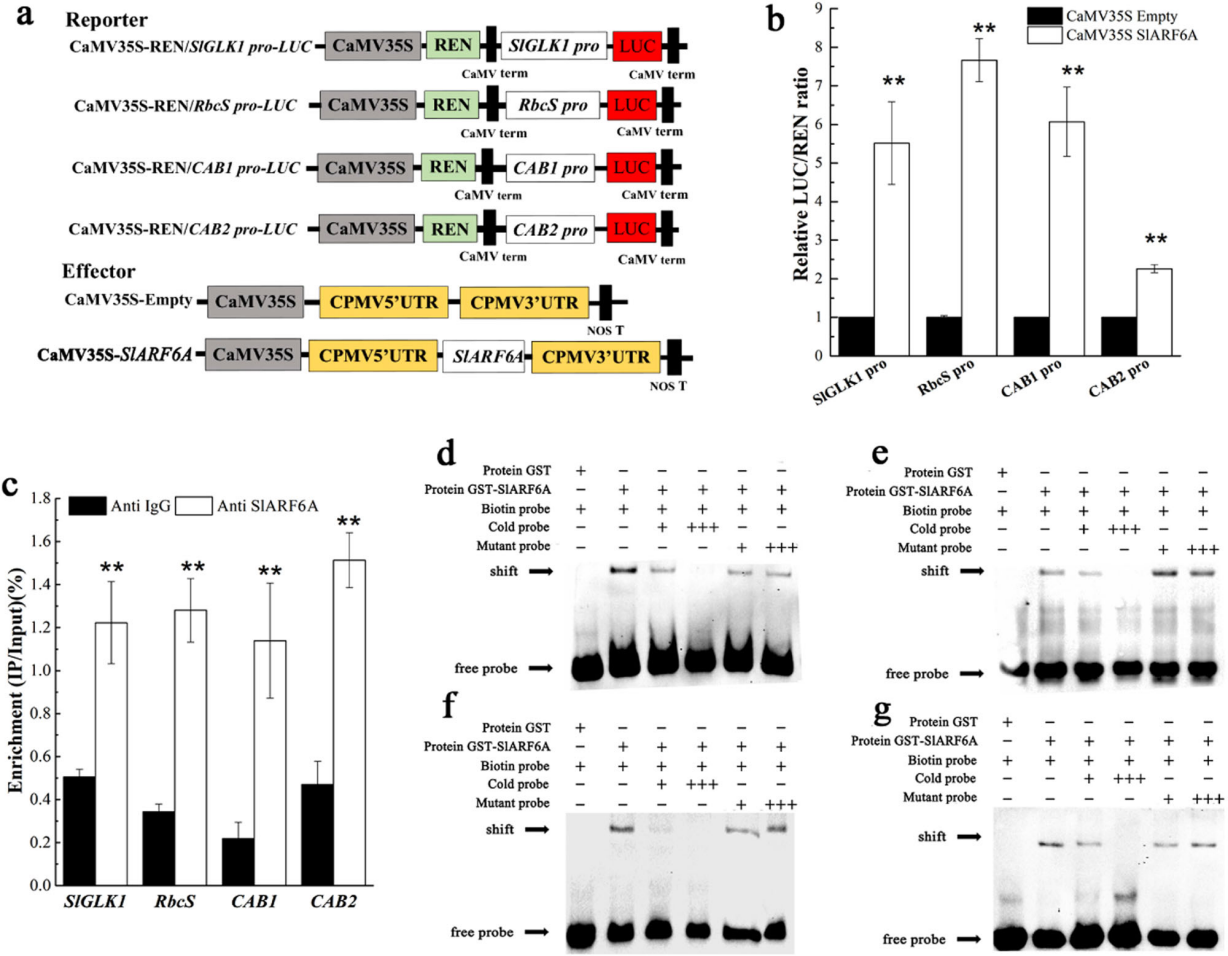
SlARF6A binds to the promoters of SlGLK1, CAB and RbcS genes and promotes the transcription of these genes. **a** Diagrams of the reporter and effector constructs used in the dual-luciferase reporter assay. **b** In vivo interactions of SlARF6A with the promoters obtained from transient assays in tobacco leaves. The ratio of LUC/REN of the empty vector plus promoter was used as a calibrator (set as 1). Each value represents the mean ± SD of six biological replicates. **c** ChIP-qPCR assay for direct binding of SlARF6A to the promoters. Values are the percentage of DNA fragments that coimmunoprecipitated with anti-FLAG antibodies or nonspecific antibodies (anti-IgG) relative to the input DNA. The data represent the mean ± SD of four biological replicates. **d**, **e**, **f**, **g** EMSA showing the binding of SlARF6A to the promoters of *CAB1*, *CAB2*, *RbcS*, and *SlGLK1*, respectively. Biotin-labeled DNA probes from native promoters or mutants were incubated with GST-SlARF6A protein, and the DNA-protein complexes were separated on 6% native polyacrylamide gels

Analysis of the promoter sequences in the *CAB* and *RbcS* genes revealed conserved ARF binding sites and TGTCTC boxes. In addition, the chlorophyll phenotypes of *SlARF6A* overexpression fruits were similar to those in *SlGLK* overexpressing lines, and the *SlGLK1* promoter contained two TGTCTC motifs. qRT-PCR identified that *SlARF6A* overexpression induced the expression of *SlGLK1* and *SlGLK2*, and knockdown of *SlARF6A* decreased the expression levels of *SlGLK1* and *SlGLK2* in fruits and leaves (Fig. S3). Dual-luciferase (LUC) reporter transient expression assays were conducted to examine whether SlARF6A could directly activate or suppress the expression of *CAB*, *RbcS*, and *SlGLK1* genes. Tobacco leaves were cotransformed with LUC reporter vectors driven by the promoters of *CAB*, *RbcS* and *SlGLK1* genes together with effector vectors carrying the CaMV35S promoter-driven *SlARF6A* gene. The results showed that LUC/REN ratios were significantly increased compared with those in the control (Fig. 7a, b). The binding of SlARF6A with the promoters in vivo was verified by ChIP-qPCR analysis. As expected, the promoter sequences containing a motif of TGTCTC in the *CAB*, *RbcS* and *SlGLK1* genes were significantly enriched with anti-SlARF6A compared with the negative control anti-IgG (Fig. 7c). Furthermore, the direct binding of SlARF6A protein to the promoters of *CAB*, *RbcS*, and *SlGLK1* was verified by an electrophoretic mobility shift assay (EMSA). We generated a recombinant glutathione S-transferase (GST) fusion protein with truncated SlARF6A (GST-tSlARF6A) (Fig. S4). The purified GST-tSlARF6A fusion protein bound to biotin-labeled probes containing the TGTCTC motif from the promoters of *CAB*, *RbcS*, and *SlGLK1* and caused a mobility shift. When unlabeled promoter fragments were used as competitors, the mobility shift was efficiently abrogated in a dose-dependent manner (Fig. 7d–g). In addition, as a negative control, the mobility shift was also abolished when biotin-labeled probes were incubated with GST only (Fig. 7d–g). This result demonstrated that SlARF6A targets the promoters of *CAB*, *RbcS*, and *SlGLK1* genes and positively regulates chlorophyll accumulation, chloroplast development and photosynthesis.

### SlARF6A directly targets the *SAMS1* promoter and negatively regulates *SAMS1* expression

**Fig. 8:**
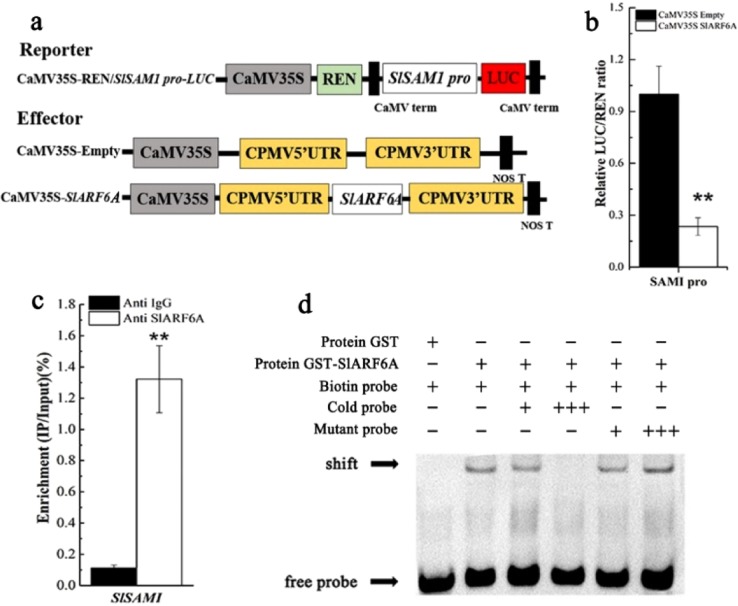
SlARF6A binds to the SAMS1 promoter and inhibits SAMS1 transcription. **a** Diagrams of the reporter and effector constructs used in the dual-luciferase reporter assay. **b** In vivo interactions of SlARF6A with the promoter obtained from transient assays in tobacco leaves. The ratio of LUC/REN of the empty vector plus promoter was used as a calibrator (set as 1). Each value represents the mean ± SD of six biological replicates. **c** ChIP-PCR assay for direct binding of SlARF6A to the *SAMS1* promoter. Values are the percentage of DNA fragments that coimmunoprecipitated with anti-FLAG antibodies or nonspecific antibodies (anti-IgG) relative to the input DNA. The data represent the mean ± SD of four biological replicates. **d** EMSA showing the binding of SlARF6A to the *SAMS1* promoter

SAMS1 is the key enzyme catalyzing the synthesis of SAM in the ethylene biosynthesis pathway. Motif analysis showed that the *SAMS1* promoter contains the conserved ARF binding site, the TGTCTC box. The transient expression assays showed that the LUC/REN ratios were significantly decreased compared with that of the control, suggesting that SlARF6A negatively regulates the expression of *SAMS1* genes (Fig. 8a, b). ChIP-qPCR was carried out to confirm the binding of SlARF6A with the *SAMS1* promoter in vivo, and the results showed that the promoter sequences containing the TGTCTC of *SAMS1* were significantly enriched compared with those with the negative control anti-IgG (Fig. 8c).

The direct binding of SlARF6A protein to the *SAMS1* promoter was further verified by EMSA. The results indicated that the SlARF6A protein directly bound to the TGTCTC motif in the *SAMS1* promoter (Fig. 8d). Taken together, SlARF6A can target the *SAMS1* promoter and negatively regulate the expression of *SAMS1* genes. The data demonstrate that SlARF6A plays an important role in ethylene production and fruit ripening.

## Discussion

In this study, we functionally characterized the transcription factor *SlARF6A* in tomato. However, there are two very similar *SlARF6* genes in the tomato genome, namely, *SlARF6A and SlARF6B*. We also examined the function of *SlARF6B* using genetic approaches and found no obvious phenotypes in the transgenic RNAi and overexpression tomato plants (data not shown). This may be related to the fact that *SlARF6B* lacks the AUX/IAA domain in the C-terminus of the protein (Fig. S1).

### *SlARF6A* regulates photosynthesis in tomato

Previous studies reported that chlorophyll accumulation increased in Arabidopsis roots when they were detached from shoots, which was repressed by auxin treatment^38^. Mutant analyses showed that auxin inhibits the accumulation of chlorophyll through the function of *IAA14*, *ARF7*, and *ARF19* in Arabidopsis^38^. In tomato, *SlARF4* plays an important role as an inhibitor in chlorophyll biosynthesis and sugar accumulation via transcriptional inhibition of *SlGLK1* expression in tomato^33,34^. In this study, overexpression of *SlARF6A* resulted in enhanced chlorophyll accumulation and chloroplast development, whereas downregulation of *SlARF6A* decreased chlorophyll accumulation and chloroplast number in tomato (Fig. 3). These results demonstrate that *SlARF6A* positively regulates chlorophyll accumulation and chloroplast number in tomato. Our study also showed that SlARF6A directly targeted the *SlGLK1* promoter and positively regulated *SlGLK1* expression (Fig. 7). Nguyen et al. (2014) reported that overexpression of *SlGLK1* and *SlGLK2* produced dark-green fruits and increased chlorophyll accumulation and chloroplast development^8^. The fact that the phenotypes of *SlGLK1* overexpression plants resembled those described in the OE-SlARF6A plants further suggests that SlARF6A positively regulates *SlGLK1* to improve chlorophyll accumulation and chloroplast development in tomato leaves and fruits.

Although *SlGLK1* and *SlGLK2* have similar functions, *SlGLK1* functions largely in leaves, while *SlGLK2* functions in fruits^8^. However, *SlGLK2* does not account for the chlorophyll phenotypes in OE and RNAi-SlARF6A plants because the ‘Micro-Tom’ variety possesses two null alleles of *SlGLK2*^39^. In our study, downregulation of *SlARF6A* reduced *SlGLK1* expression and chlorophyll accumulation, whereas overexpression of *SlARF6A* increased *SlGLK1* expression and chlorophyll accumulation in leaves and fruits of tomato plants (Figs. 2 and 3). The data demonstrate that *SlGLK1* may be involved in chlorophyll accumulation not only in tomato leaves but also in fruits. Further study is needed to elucidate the important role of *SlGLK1* in tomato fruit using CRISPR/Cas9 technologies.

The chlorophyll a/b-binding proteins (CABs) are the apoproteins of the light-harvesting complex of photosystem II (PSII). CABs are normally complexed with xanthophylls and chlorophyll, functioning as the antenna complex, and are involved in photosynthetic electron transport^40^. Meng et al. (2018) reported that SlBEL11 directly acted on the promoter of *CABs* to suppress their transcription^10^. Silencing of *SlBEL11* increased the expression of *CAB* genes, resulting in enhanced chlorophyll accumulation and stability in thylakoid membranes of chloroplasts in green tomato fruit^10^. In our study, SlARF6A targeted the promoter of *CABs*, which positively regulated chlorophyll accumulation, chloroplast development and photosynthesis in tomato (Figs. 2, 3, 4 and 6). Our data further demonstrate important roles of CABs in chloroplast activity and photosynthesis in tomato.

Rubisco, a key enzyme in the fixation of CO_2_, is the rate-limiting factor in the photosynthesis pathway under conditions of saturating light and atmospheric CO_2_^41^. The *RbcL* and *RbcS* genes encode two subunits that form the Rubisco enzyme^42^. The *RbcL* and *RbcS* genes are localized to the chloroplasts and to the nucleus, respectively^43^. Our study showed that overexpression of *SlARF6A* increased the expression of the *RbcS* gene. Moreover, SlARF6A directly targeted the *RbcS* promoter and positively regulated *RbcS* expression (Fig. 7). In addition, *SlARF6A* positively affected photosynthesis in the fruits and leaves of tomato plants (Fig. 4). Our study demonstrates that *SlARF6A* has important roles in photosynthesis via the direct regulation of the *RbcS* gene in tomato.

Interestingly, RNA-Seq data showed that the expression levels of *SlARF4* and *SlARF10* genes were not altered in RNAi-SlARF6A and OE-SlARF6A plants, suggesting that SlARF6A may act on chlorophyll accumulation independently of SlARF4 and SlARF10. However, studies indicate that ARFs must form dimers on palindromic TGTCTC AuxREs to form a stable complex, leading to the possibility that SlARF6A, SlARF4 and SlARF10 could form dimers with each other to regulate chlorophyll metabolism^27^. Further study could focus on the interactions among SlARF6A, SlARF4, and SlARF10 to comprehensively elucidate the effects of the transcriptional regulation of ARFs on chlorophyll accumulation in tomato.

### *SlARF6A* regulates photosynthate accumulation in tomato

Downregulation of *SlARF4* increased the photosynthesis rate and enhanced the accumulation of starch, glucose and fructose in tomato fruits^8^. In this study, the increased chlorophyll accumulation and photosynthesis rate in OE-SlARF6A plants resulted in the increased contents of starch and soluble sugars in fruits (Fig. 4). Starch is a dominant factor in the nutrients and flavor of fruits^8^. AGPase catalyzes the first regulatory step in starch synthesis, converting glucose-1-phosphate and ATP into ADP-glucose^44,45^. This critical catalytic reaction is also a limiting step during starch biosynthesis in potato (*Solanum tuberosum*) tubers^46^. Knockdown of *SlARF4* increases the expression of *AGPase* genes and starch content^8^. In this study, *SlARF6A* was positively correlated with the expression of *AGPase* genes (Fig. S3), suggesting the important role of *AGPase* genes in starch biosynthesis in tomato. However, the EMSA failed to detect any binding between SlARF6A and the promoters of *AGPase* genes, even though auxin-responsive motifs were detected in the promoters of *AGPase S1* and *AGPase S2* genes.

Evidence suggests that sucrose induces the expression of *AGPase* genes in leaves and fruits in tomato^47^. In this study, overexpression of *SlARF6A* led to increased sucrose content in tomato fruits, while the RNAi-SlARF6A fruits displayed decreased sucrose accumulation (Fig. 4). The altered accumulation of starch in OE-SlARF6A and RNAi-SlARF6A lines may be explained by the altered expression of *AGPase* genes influenced by sucrose in tomato. Overexpression of *SlARF6A* also resulted in increased glucose and fructose content, which was likely due to the increased starch content degraded into increased contents of soluble sugars in tomato fruits. Our results are consistent with the notion that incipient starch content determines soluble sugars in the process of fruit development^48,49^. Our study also provides a valuable method to improve the nutritional value of tomato fruits via regulation of *SlARF6A* expression.

### *SlARF6A* is involved in ethylene production and fruit ripening in tomato

The tomato *ARF2A* gene was reported to positively regulate fruit ripening^50^. Overexpression of *ARF2A* in tomato resulted in blotchy ripening, and silencing of *ARF2A* led to retarded fruit ripening^50^. Overexpression of *ARF2A* in tomato promoted early production of ethylene and expression of ethylene biosynthesis and receptor genes. In this study, *SlARF6A* negatively regulated fruit ripening and ethylene biosynthesis in tomato fruit (Fig. 5). S-adenosyl-L-methionine (SAM), synthesized by SAM synthetase from ATP and methionine, is a substrate for ethylene biosynthesis (Roje, 2006). SAM is converted to ACC by the ACS enzyme, and ACC is then converted to ethylene by ACO^51,52^. The level of SAM is tightly controlled to integrate developmental signals into the hormonal control of plant development^47,53^. In Arabidopsis, overexpression of *SAMS1* increases the SAM and ethylene levels, whereas sam1/2 mutants show the opposite phenotype in seedlings^54^. Similarly, in tomato plants, overexpression of *SAMS1* results in higher concentrations of ACC and ethylene compared with those in WT plants^55^. These data indicate the important role of the *SAMS1* gene in ethylene biosynthesis in plants. In this study, SlARF6A directly targeted the *SAMS1* promoter and negatively regulated *SAMS1* expression (Fig. 8). The regulatory mechanism by which *SlARF6A* affects fruit ripening and ethylene production in tomato fruits can be explained by the interaction between SlARF6A and the *SAMS1* promoter.

It is interesting that ethylene and auxin interact with each other to control some plant developmental processes. For example, ethylene controls root growth through regulation of auxin biosynthesis, transport and signaling^56,57^, while the formation of hypocotyl apical hooks is also regulated in a similar fashion in Arabidopsis^58^. In tomato, knockdown of *IAA3* results in both auxin and ethylene phenotypes, suggesting that *IAA3* might be the molecular connection between ethylene and auxin^59^. Liu et al. (2018) reported that the ethylene response factor SlERFB3 integrated ethylene and auxin signaling through direct regulation of the *Aux/IAA27* gene in tomato^59^. Our results indicate that SlARF6A negatively regulates ethylene biosynthesis and that the interaction of SlARF6A and *SAMS1* represents an important integrative hub mediating ethylene-auxin cross-talk in tomato.

In summary, our results demonstrate that SlARF6A regulates chlorophyll level and chloroplast development by directly binding to the promoters of the *SlGLK1*, *CAB1*, and *CAB2* genes. SlARF6A also directly targets the *RbcS* gene promoter, activating *RbcS* expression and increasing the photosynthetic rate. The increased chlorophyll accumulation and chloroplast activity improve photosynthesis, resulting in the increased accumulation of starch and soluble sugars in tomato. In addition, SlARF6A can act directly on the promoter of *SAMS1* and negatively regulate its expression, thereby influencing ethylene production and fruit ripening. The present study provides new insight into the link between auxin signaling, chloroplast activity, and ethylene biosynthesis during tomato fruit development. Our data also provide an effective way to improve fruit nutrition of horticulture crops via regulation of chlorophyll accumulation and photosynthetic activity.

## Materials and methods

### Plant materials and growth conditions

Tomato plants (*Solanum lycopersicum* ‘Micro-Tom’) were used in this study. ‘Micro-Tom’ is a popular variety because of its fast turnaround time and easy transformation. The plants were grown on soil under standard greenhouse conditions with a 14-h-day/10-h night cycle, 25 °C/20 °C day/night temperature, 60% relative humidity and 250 mol m^–2^ s^–1^ intense light. Transgenic seeds of T1, T2 and T3 generations were screened by sterilizing, rinsing in sterile water, and then transfer into Magenta vessels containing 40 mL of 1/2-strength Murashige and Skoog medium with R3 vitamin (100 mg L^–1^ kanamycin, 0.5 mg L^–1^ thiamine, 0.5 mg L^–1^ pyridoxine and 0.25 mg L^–1^ nicotinic acid), 0.8% (w/v) agar, and 1.5% (w/v) sucrose, pH 5.9.

### Plasmid construction and generation of transgenic plants

DNA fragments, the *SlARF6A* (Solyc12g006340) promoter, the full-length *SlARF6A* coding sequence and a partial *SlARF6A* coding sequence were amplified using tomato genomic DNA or cDNA. The PCR primers used for amplification are detailed in Supplementary Table S3. The *SlARF6A* promoter sequence was cloned into a pLP100 vector containing the *GUS* reporter gene. To obtain overexpressed *SlARF6A* vector, the ORF sequence of *SlARF6A* was cloned into plant binary vector pLP100 in the sense orientation under the transcriptional control of a cauliflower mosaic virus (CaMV) 35 S promoter. For construction of the RNAi vector, the 200 bp sequences of *SlARF6A* were amplified and inserted in pCAMIBA2301 under control of the (CaMV) 35S promoter and a nopaline synthase terminator. The resulting transgenic plant was obtained by *Agrobacterium tumefaciens*-mediated transformation according to Jones et al. (2002)^33^. All experiments were performed using homozygous lines from the T3 generation.

### qRT-PCR analysis

Tomato total RNA was extracted using an RNeasy Plant Mini Kit (Qiagen, Valencia, CA, USA), and qRT-PCR was carried out using All-in-One™ qPCR Mix (GeneCopoeia, Rockville, MD, USA) with a CFX96 real-time PCR detection system (Bio-Rad, Hercules, CA, USA) according to Zhang et al. (2015)^32^. The relative expression levels of genes were calculated from ΔΔCt values using ubiquitin gene expression as an internal control. The primer sequences used for qRT-PCR are listed in Supplementary Table S3.

### GUS staining and analysis

Tissues from *SlARF6A* promoter-*GUS* plants were collected and submerged in GUS staining solution (0.1 M sodium phosphate buffer, pH 7.2, 10 mM EDTA). After being infiltrated with GUS staining solution under vacuum for 15 min twice, tissues were incubated in the solution at 37 °C overnight. Then, the samples were washed via a graded ethanol series and observed under a light microscope.

### Subcellular localization and transcriptional activation activity of SlARF6A

The ORF sequence of *SlARF6A* was cloned into a PCX-DG vector to generate the *SlARF6A*-*GFP* fusion expression vector. Specific primer sequences are listed in Supplementary Table S1. Suspension-cultured tobacco (*Nicotiana tabacum* cv. Bright Yellow-2) cells were used to obtain protoplasts that were transfected with the *SlARF6A*-*GFP* fusion expression vector. Transformation assays were carried out according to the procedures described by Chaabouni et al. (2009)^60^.

The ORF sequence of *SlARF6A* was amplified and fused to the GAL4 DNA-binding (DB) domain to obtain the pGBKT7-*SlARF6A* fusion construct (DB-*SlARF6A*). The pGBKT7-*SlARF6A* vectors were transformed into Y2H gold yeast cells and cultivated on plates in minimal medium without tryptophan (SD-Trp) or without tryptophan, histidine, and adenine (SD-Trp/His/Ade). The transcriptional activation activity was analyzed based on the growth status and α-galactosidase (α-gal) activity.

### Chlorophyll analysis and chlorophyll fluorescence parameter measurements

For chlorophyll content determination, the fruits at different developmental stages and leaf tissues were collected and examined based on the methods described by Powell et al. (2012)^39^. To determine chlorophyll autofluorescence, pericarp was peeled off tomato fruits and observed with a TCS SP2 laser confocal microscope (Leica, Germany). For transmission electron microscopy, pericarp tissues were examined with an FEI Tecnai T12 twin transmission electron microscope according to the method described by Nguyen et al. (2014)^8^.

For measurements of photosynthesis rates, the green mature fruits and leaves were measured via a PAM-2500 pulse-amplitude modulation fluorometer (Heinz Walz, Effeltrich, Germany). The chlorophyll fluorescence parameter was measured based on the method described by Maury et al. (1996)^61^.

### Extraction and assay of metabolites

For sugar extraction, 1 g of fruit tissue was collected and ground under liquid nitrogen. Subsequently, 10 mL of 80% (v/v) ethanol was used for extraction three times at 80℃ for 30 min. After centrifugation, samples were completely evaporated under vacuum and then dissolved in 4 mL of distilled water. Using the dissolved samples, HPLC was carried out to determine the content of sucrose, fructose and glucose. Starch content determination was performed using fruit pellets. Four milliliters of 0.2 M KOH was used to dissolve the pellet by incubating the sample in a boiling water bath for 30 min. Then, 1.48 mL of 1 M acetic acid (pH 4.5) with 7 units of amyloglucosidase was employed to hydrolyze each sample for 45 min. Finally, 10 mL of distilled water was adopted to dissolve the sample, and then the dissolved sample was used for starch content measurement.

For metabolite measurement, HPLC analysis was conducted using an Agilent 1260 Series liquid chromatograph system (Agilent Technologies, Palo Alto, CA, USA) with a vacuum degasser, an autosampler, a binary pump, and a diode array detector (DAD) controlled by Agilent ChemStation software. A precolumn (Waters XBridge BEH Amide column, 3.9 × 5 mm i.d., 3.5 μm) and a Waters XBridge Amide column (4.6 × 150 mm i.d., 3.5 μm) were used for analysis. The separation was performed via an isocratic solvent system with solvent A (0.2% triethylamine water solution) and solvent B (acetonitrile), while the mobile phase was maintained at 75% B for 15 min for elution. The column temperature was maintained at 38 °C, and the solvent flow rate was 0.6 mL/min. Meanwhile, the injection volume was 10 μL for each sample. With a drift tube temperature at 80 °C, the detection system for HPLC was an ELSD 2000, and air was used as the carrier gas with a flow rate of 2.2 L/min. Finally, the contents of glucose, fructose, sucrose and starch in tomato fruits were determined based on the methods described by Geigenberger et al. (1996)^62^.

### RNA-Seq analysis

The ovaries (4 DPA) of WT and RNAi-SlARF6A plants and the mature green fruits (35 DPA) of WT and OE-SlARF6A plants were collected for RNA-Seq analysis. Total RNA was isolated using a DNeasy Plant Mini Kit (Qiagen, Valencia, CA, USA). RNA-Seq was carried out at the Shanghai Majorbio Biopharm Technology Co., Ltd., as described by Zhang et al. (2015)^32^. The Illumina HiSeq^TM^ 2000 platform was used according to the manufacturer’s instructions. All clean reads were aligned to the tomato genome (http://solgenomics.net/organism/Solanum_lycopersicum/genome) using TopHat (http://tophat.cbcb.umd). Transcript abundance was normalized by the fragments per kilobase of exon per million mapped reads (FRKM) method using Cuffdiff software (http://cole-trapnell-lab.github.io/cufflinks/). A false discovery rate (FDR) of less than 0.05 was used as the threshold for differentially expressed genes (DEGs). GO functional enrichment and KEGG pathway analysis were carried out using goatools (https://github.com/tanghaibao/goatools) and KOBAS (http://kobas.cbi.pku.edu.cn/home.do). Pathway enrichment was analyzed using the Benjamini and Hochberg correction method with FDR < 0.05.

### Promoter analysis and dual-luciferase transient expression assay

For promoter analysis, PLACE signal scan search software (http://www.dna.affrc.go.jp/PLACE/signalscan.html) was used to analyze the motifs of target genes. A dual-luciferase transient expression assay for *SlARF6A* was carried out using tobacco leaves (*Nicotiana benthamiana*). For effector vector construction, the full coding sequence of *SlARF6A* was amplified and then cloned into the pGreenII 62-SK vector^63^. For reporter vector construction, the promoters of *SlGLK1*, *CAB*, *RbcS*, and *SAMS1* genes were cloned into a pGreenII 0800-LUC vector (Hellens et al., 2005)^63^. The primer sequences used for the vector construct are shown in Supplementary Table S3. A dual-luciferase assay kit (Promega, USA) was employed to measure the activities of LUC and REN luciferase according to the manufacturer’s instructions via a Luminoskan Ascent microplate luminometer (Thermo Fisher Scientific, USA). For each pair of vectors, six biological repeats were performed.

### Protein expression and EMSA

The nucleotide sequence of the putative DNA-binding domain of *SlARF6A* (from 1 to 978 bp) was amplified and fused to that of the glutathione S-transferase (GST) tag in a pGEX-4T-1 bacterial expression vector (GE Healthcare Life Science, China) and expressed using *Escherichia coli* strain BM Rosetta (DE3). Isopropyl-β-D-thiogalactopyranoside (1 mM) was used to induce recombinant protein expression, and a GST-Tagged Protein Purification Kit (Clontech, USA) was used to purify the protein. Purified recombinant proteins and biotin-labeled fragments of the target promoters were used to conduct EMSA with a LightShift Chemiluminescent EMSA kit (Thermo Fisher Scientific, USA) based on the method described in detail by Han et al. (2016)^64^. The Pierce Biotin 3’ End DNA Labeling Kit (Thermo Fisher Scientific, USA) was employed to label the probe containing the TGTCTC sequence with biotin. The unlabeled same sequence was used in the assay as a competitor. To generate the mutant probe, the TGTCTC DNA fragment was changed to AAAAAA. Biotin-labeled DNA was assayed via a ChemiDoc™ MP Imaging System (Bio-Rad, USA) based on the manufacturer’s procedures. All primers for the EMSA are listed in Supplementary Table S3.

### Chromatin immunoprecipitation

A ChIP-qPCR assay was carried out based on the method described in detail by Qin et al. (2012)^65^. All primer sequences used in this analysis are listed in Table S3.

## Supplementary information

Supplementary figures

Table S3

Table S1

Table S2
