# Supplementary material for: Auxin response factor 6A regulates photosynthesis, sugar accumulation, and fruit development in tomato
Source: Hortic Res. 2019 Jul 11;6:85. doi: 10.1038/s41438-019-0167-x (PMC6804849; doi:10.1038/s41438-019-0167-x)
Supplement: Supplementary file 1 — Supplementary figures [file 41438_2019_167_MOESM1_ESM.docx]

**
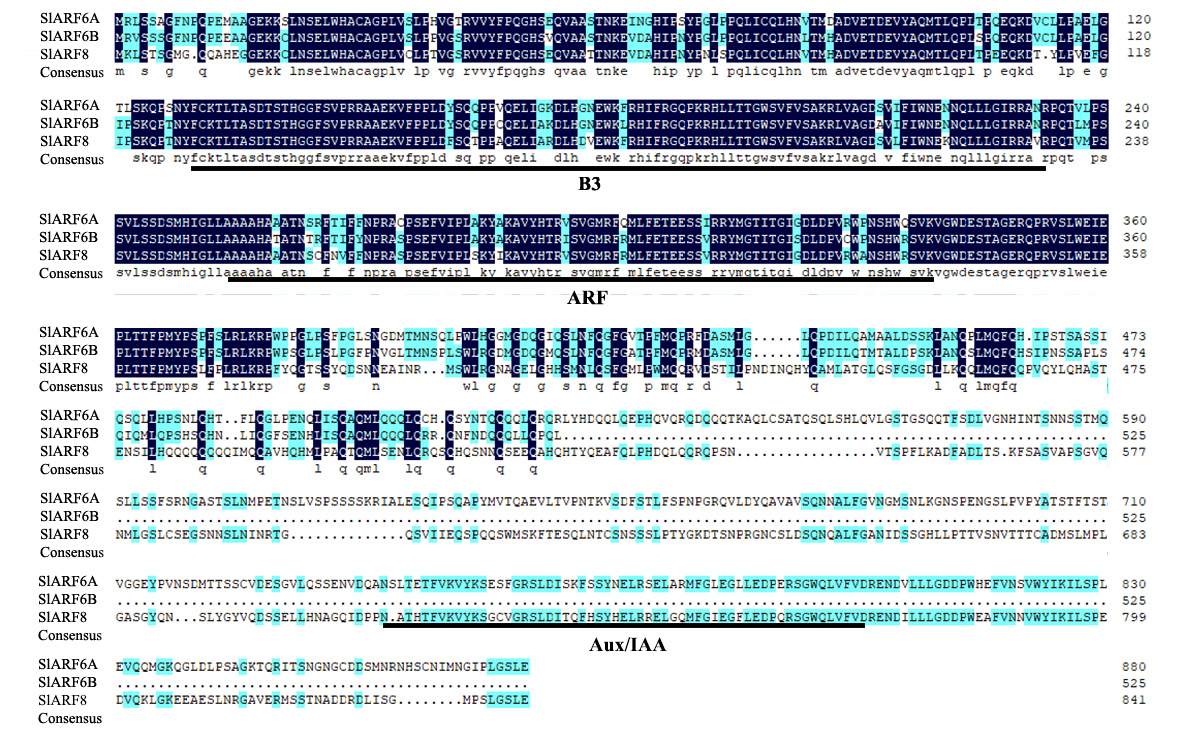
**

**

Fig. S1.** Sequence comparison of SlARF6A, SlARF6B and SlARF8. SlARF6B doesn’t have Aux/IAA domain.

**
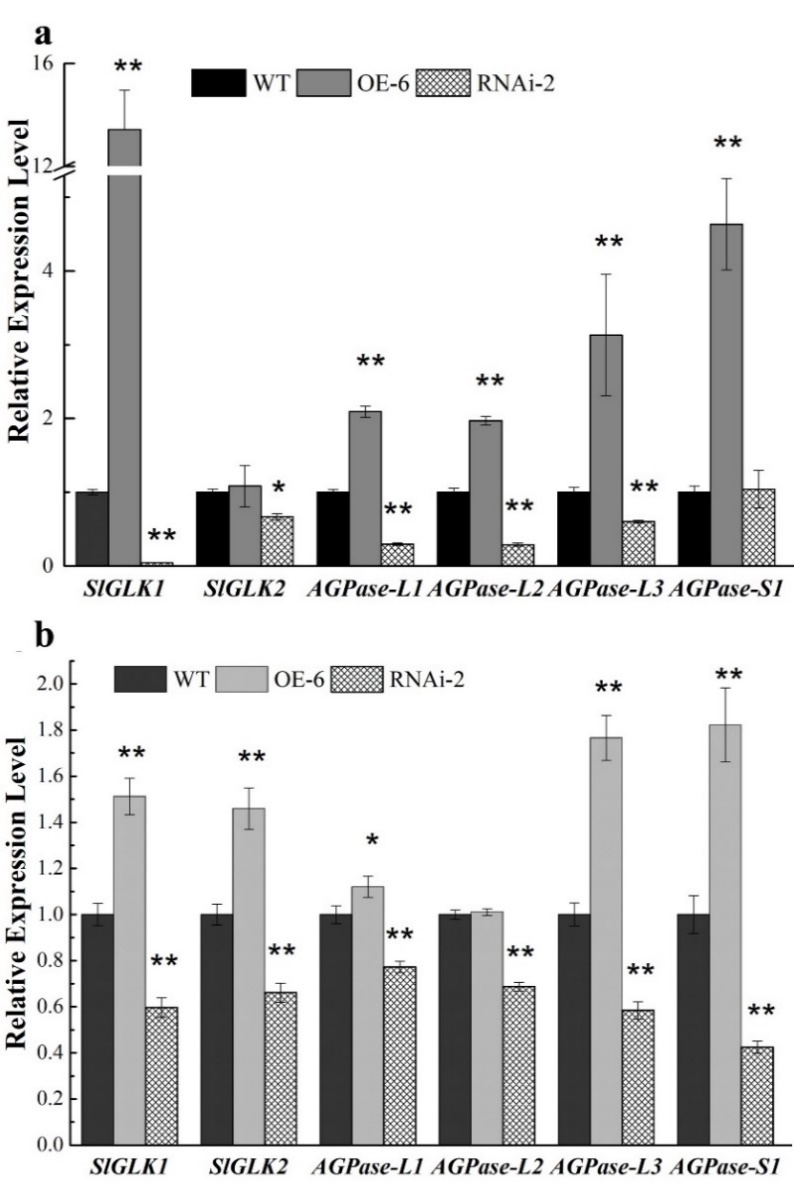
Fig. S2.** Phylogenetic analysis of SlARFs and AtARFs.

**Fig. S3.** qRT-PCR analysis of the expression of *SlGLK1, SlGLK2, AGPase L1, AGPase L2, AGPase L3* and *AGPase S1* in leaves (a) and fruits (b) of *SlARF6A* transgenic lines. The data represent mean ±SD of four biological replicates. OE-6, *SlARF6A* overexpression lines. RNAi-2, *SlARF6A* RNAi lines. “*” and “**” are signiﬁcant differences between transgenic and WT plants at P <0.05 and P <0.01, respectively, as determined by t-test.

**
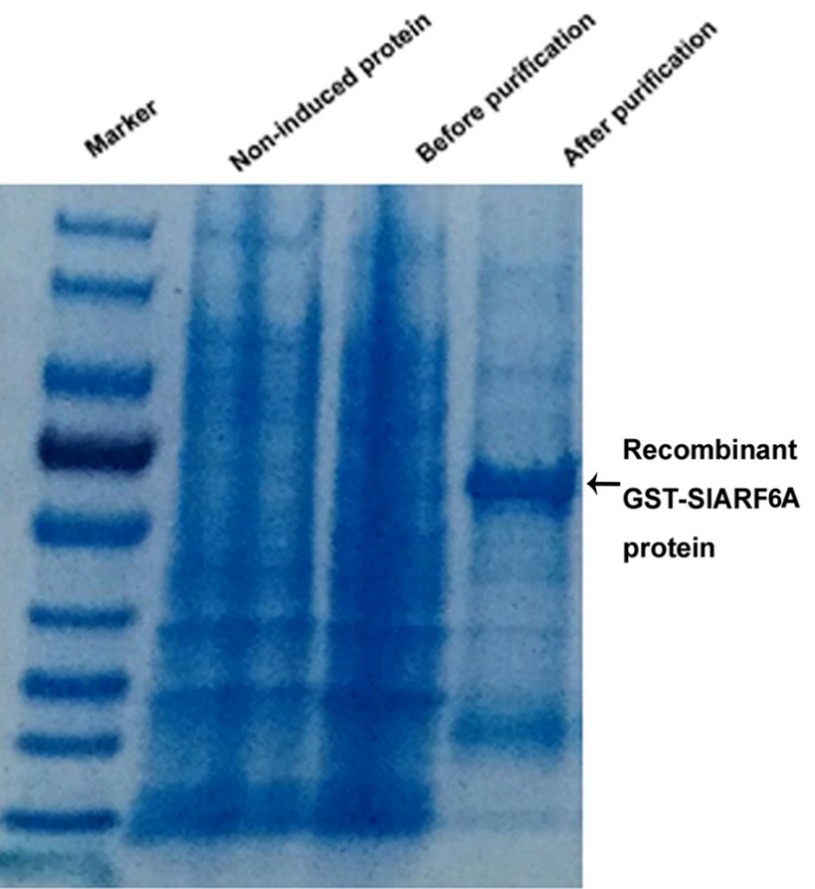
**

**Fig. S4**. SDS-PAGE gel stained with coomassie brilliant blue demonstrating affinity purification of the recombinant GST-SlARF6A protein used for the EMSA assay.

**Table S1**. DEGs in SlARF6A plants.

**Table S2**. GO function and pathway enrichment analyses

**Table S3**. Primers used for qRT-PCR, vector construction and EMSA.
